# Preoperative core needle biopsy is accurate in determining molecular subtypes in invasive breast cancer

**DOI:** 10.1186/1471-2407-13-390

**Published:** 2013-08-19

**Authors:** Xiaosong Chen, Long Sun, Yan Mao, Siji Zhu, Jiayi Wu, Ou Huang, Yafen Li, Weiguo Chen, Jianhua Wang, Ying Yuan, Xiaochun Fei, Xiaolong Jin, Kunwei Shen

**Affiliations:** 1 Comprehensive Breast Health Center, Ruijin Hospital, Shanghai Jiaotong University School of Medicine, 197 Ruijin Second Road, Shanghai 200025, China; 2 Department of Biochemistry and Molecular & Cell Biology, Shanghai Jiaotong University School of Medicine, 227 Chongqing Nan Road, Shanghai 200025, China; 3 Department of Radiology, Shanghai Ninth People’s Hospital, Affiliated to Jiaotong University School of Medicine, 639 Zhizhaoju Road, Shanghai 200011, China; 4 Department of Pathology, Ruijin Hospital, Shanghai Jiaotong University School of Medicine, 197 Ruijin Second Road, Shanghai 200025, China

**Keywords:** Breast cancer, Core needle biopsy, Molecular subtype, Ki67, Concordance rate

## Abstract

**Background:**

Estrogen receptor (ER), progesterone receptor (PgR), HER2, and Ki67 have been increasingly evaluated by core needle biopsy (CNB) and are recommended for classifying breast cancer into molecular subtypes. However, the concordance rate between CNB and open excision biopsy (OEB) has not been well documented.

**Methods:**

Patients with paired CNB and OEB samples from Oct. 2009 to Feb. 2012 in Ruijin Hospital were included. ER, PgR, HER2, and Ki67 were determined by immunohistochemistry (IHC). Patients with HER2 IHC 2+ were further examined by FISH. Cutoff value for Ki67 high expression was 14%. Molecular subtypes were constructed as follows: Luminal A, Luminal B, Triple Negative, and HER2 positive.

**Results:**

There were 298 invasive breast cancer patients analyzed. Concordance rates for ER, PgR, and HER2 were 93.6%, 85.9%, and 96.3%, respectively. Ki67 expression was slightly higher in OEB than in CNB samples (29.3% vs. 26.8%, *P* = 0.046). Good agreement (κ = 0.658) was demonstrated in evaluating molecular subtypes between CNB and OEB, with a concordance rate of 77.2%. We also used a different Ki67 cutoff value (20%) for determining Luminal A and B subtypes in HR (hormone receptor) +/HER2- diseases and the overall concordance rate was 79.2%. However, using a cut-point of Ki67 either 14% or 20% for both specimens, there will be about 14% of HR+/HER2- specimens that are called Luminal A on CNB and Luminal B on OEB.

**Conclusion:**

CNB was accurate in determining ER, PgR, and HER2 status as well as non-Luminal molecular subtypes in invasive breast cancer. Ki67 should be retested on OEB samples in HR+/HER2- patients to accurately distinguish Luminal A from B tumors.

## Background

Breast cancer is the most common malignancy affecting women. However, the mortality has decreased in western countries due to earlier diagnosis and more comprehensive treatment [1]. The core needle biopsy (CNB) procedure is almost as accurate as an open excision biopsy (OEB) in the diagnosis of breast diseases, and is now widely taken as the standard procedure for a breast cancer diagnosis [2]. The 2011 European Society of Medical Oncology breast cancer clinical practice guideline required a preoperative disease-related staging, including pathological examination of the CNB with a report on estrogen receptor (ER), progesterone receptor (PgR), and human epidermal growth factor receptor-2 (HER2) status by immunohistochemistry (IHC) or fluorescence in situ hybridization (FISH) [3]. A recent meta-analysis showed that CNB tissue could replace OEB for determining ER, PgR, and HER2 status [4]. Breast cancer is a heterogeneous disease and microarray expression data have demonstrated that there are at least four subtypes of breast cancer, including Luminal A, Luminal B, HER2positive, and basal-like subtypes [5]. Practically, these subtypes can be approximated using clinicopathological markers rather than gene expression array criteria. The 2011 St.Gallen breast cancer consensus also recommended that the IHC status of ER, PgR, HER2, and Ki67 could be used to approximately classify breast cancer into these subtypes, which can guide subsequent systemic treatment [6]. However, due to its relatively smaller sample size and tumor heterogeneity, the biomarker assessment performed on CNB samples may be less reliable than in OEB [7-9]. Little has been reported on the comparison of molecular breast cancer subtype between CNB and OEB.

Therefore, using IHC and FISH to detect the ER, PgR, HER2, and Ki67 status in CNB and subsequent OEB samples, we then constructed breast cancer molecular subtypes. Our aim was to estimate the concordance between CNB and OEB in evaluating molecular subtypes as well as the receptor status and Ki67 expression levels.

## Methods

### Patient population and samples

We retrospectively and consecutively analyzed patients with paired CNB and OEB samples from Oct. 2009 to Feb. 2012 in Ruijin Hospital, Shanghai Jiaotong University School of Medicine, Shanghai, China. All CNB were performed under ultrasound guidance, with at least four 14-gauce core biopsies being obtained for pathological examination. Patients who met all the following criteria were included: (1) received both CNB and OEB in our center; (2) found invasive carcinoma in both CNB and OEB samples; (3) female gender; (4) no preoperative therapy; (5) samples available for IHC and FISH analysis; (6) HER2 IHC 2+ result further confirmed by FISH test. The study was conducted in accordance with the Declaration of Helsinki. The protocol was reviewed and approved by the independent Ethical Committee/Institutional Review Board of Ruijin Hospital, Shanghai Jiaotong University School of Medicine, Shanghai, China.

### Receptor status evaluation and molecular subtypes classification

**Figure 1:**
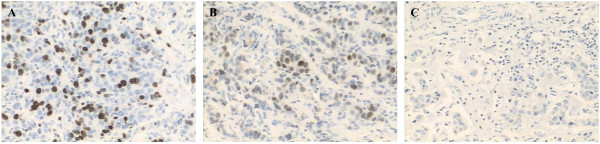
**Ki67 expression in breast cancer. A**: positive Ki67 nuclei; **B**: faintly positive Ki67 nuclei; **C**: negative Ki67 nuclei.

IHC assessment of ER (SP1, DAKO), PgR (PgR 636, DAKO), Ki67 (MIB-1, DAKO) and HER2 (4B5, Roche) were made from paraffin-embedded tumor samples from CNB and OEB by Ventana autostain system, BenchMark XT, and evaluated with internal and positive controls. All IHC and FISH results were firstly retrospective collected and then further reviewed by two senior pathologists (Xiaochun Fei, and Xiaolong Jin, who diagnosed more than 300 breast cancer patients per year and achieved as high as 90% concordance rate in evaluating these IHC or FISH results) for this study purpose. ER-positivity (ER+) and PgR-positivity (PgR+) were defined as more than 1% positive invasive tumor cells with nuclear staining [10]. HER2 was firstly determined by IHC and scored as 0 to 3+ according to ASCO/CAP (American Society of Clinical Oncology/College of American Pathologists) guideline [11]. Samples with IHC HER2 2+ were further examined by FISH and the tumor was considered to have HER2 amplification if the ratio of HER2 gene signals to chromosome 17 signals was ≥ 2.2. Tumors with HER2 IHC 3+ or FISH + were regarded as HER2 positivity (HER2+). For Ki67 expression scoring, we firstly reviewed the cell distribution over the whole slice and used the same method for scoring CNB and OEB samples. If Ki67 expression was uniformly distributed over the entire slide, 500–2000 cells were chosen from different microscope views; otherwise, 2000 cells were equally counted in both hotspot and negative areas in slice. Ki67 expression was scored as the percentage of positive invasive tumor cells with any nuclear staining and recorded as mean percentage of positive cells (Figure 1) [12]. All IHC and FISH analyses were conducted in the Department of Pathology, Ruijin Hospital, Shanghai Jiaotong University School of Medicine, which participated in an external quality control program and classified as “excellent” quality by WHO-British UKNEQAS (United Kingdom National External Quality Assessment Service) organization.

Hormonal receptor positivity (HR+) was defined as either ER + or PgR+, and HR– as both ER– and PgR–. To determine the Luminal status in HR+/HER2- tumors, the cutoff value of Ki67 high expression was set as 14%. Thus, there were four breast cancer subtypes as classified according to the 2011 St. Gallen breast cancer consensus [6]: Luminal A (HR+/HER2–, Ki67 low), Luminal B (HR+/HER2-, Ki67 high or HR+/HER2+), triple negative (HR-/HER2–) and HER2 positive (HR-/HER2+). We further subdivided our Luminal B cases into Luminal B-HER2- (HR+/HER2-, Ki67 high) and Luminal-HER2+ (HR + and HER2+) subtypes. To mimic the actual and convenient clinical practice situation, we also used 20% as Ki67 cutoff value to classify Luminal A and B subtypes, which was the mean value for HR+/HER2- patients and median value for the whole patients in CNB samples.

### Statistical analysis

Concordance analysis of receptor status and molecular subtypes was performed on CNB and OEB samples. Statistical analysis, including positive and negative agreement, was calculated using kappa test. Values of κ > 0.6 were correlated with good agreement, values between 0.4 and 0.6 were considered as moderate agreement, values <0.4 as fair, and values <0.2 as poor agreement [13]. All statistical tests were two-sided and carried out at a significance level of 0.05 using the SPSS statistical software package (version 13.0; SPSS Company, Chicago, IL).

## Results

### Patient characteristics

There were 298 invasive breast cancer patients eligible for this study. The median age was 54 (24–91) years. Most patients received mastectomy and 55.6% were diagnosed as having negative axillary lymph nodes (Table 1).

**Table 1:** Baseline patient characteristics

**Characteristic**	**No.**	**%**
**Age, years**	54 (24-91)	
**Menstrual status**		
Peri/pre-menopause	98	32.9
Post-menopause	200	67.1
**Breast surgery type**		
Mastectomy (+/-reconstruction)	259	86.9
Lumpectomy	39	13.1
**Tumor size**		
Tx	7	2.3
≤2 cm	123	41.3
2-5 cm	160	53.7
>5 cm	8	2.7
**Axillary lymph node**		
Negative	165	55.4
1-3 +	76	25.5
≥4+	55	18.5
Nx	2	0.7

The expression rates of ER, PgR, HER2, and molecular subtypes showed no significant difference between CNB and OEB (Table 2). However, the mean Ki67 expression was slightly higher in OEB than in CNB samples (*P* = 0.046), these being 29.3% and 26.8%, respectively. Median Ki67 was 20% (1%-90%) for CNB samples in this cohort. Furthermore, in HR+/HER2- tumors, the mean Ki67 value was also 20% in CNB samples.

**Table 2:** Tumor characteristics for CNB and OEB results

**Characteristic**	**No. (%)**		**P value**
	**CNB**	**OEB**	
**Pathological type**			0.190
Invasive ductal carcinoma	260 (87.2)	269 (90.3)	
Invasive lobular carcinoma	12 (4.0)	11 (3.7)	
Mixed carcinoma	11 (3.7)	3 (1.0)	
Others	15 (5.0)	15 (5.0)	
**Histological grading**			0.009
I	6 (2.0)	4 (1.3)	
II	138 (46.3)	151 (50.7)	
III	97 (32.6)	114 (38.3)	
NA	57 (19.1)	29 (9.7)	
**Estrogen Receptor**			0.294
Negative	67 (22.5)	78 (26.2)	
Positive	231 (77.5)	220 (73.8)	
**Progesterone Receptor**			0.502
Negative	120 (40.3)	112 (37.6)	
Positive	178 (59.7)	186 (62.4)	
**HER2**			0.768
Negative	233 (78.2)	230 (77.2)	
Positive	65 (21.8)	68 (22.8)	
**Ki67 (%, mean)**	26.8 (1-90)	29.3 (1-90)	0.046
<14	112 (37.6)	89 (29.9)	
≥14	186 (62.4)	209 (70.1)	
**Hormonal receptor**			0.386
Negative	66 (22.1)	75 (25.2)	
Positive	232 (77.9)	223 (74.8)	
**Molecular subtype**			0.484*
Luminal A	97 (32.6)	80 (26.8)	
Luminal B	135 (45.4)	143 (48.0)	
Luminal B (HER2-)	97 (32.6)	105 (35.2)	
Luminal-HER2+	38 (12.8)	38 (12.8)	
Triple negative	39 (13.1)	45 (15.1)	
HER2 positive	27 (9.1)	30 (10.1)	

*Abbreviation*: CNB, core needle biopsy; OEB, open excision biopsy; NA, not available;

*: calculated with four subtypes classification.

### Comparison of CNB with OEB for receptor status and Ki67 results

Evaluation of ER expression on CNB samples had a 93.6% concordance rate with ER results on OEB samples, with good overall agreement (κ = 0.827). PgR and HER2 expression on CNB samples also revealed good agreement with those on OEB samples; the overall concordance rates were 85.9% and 96.3%, with κ value of 0.704 and 0.894, respectively (Table 3). In addition, similar to the ER and PgR results, a good agreement was observed in terms of HR detection, with a concordance rate of 93.6% (κ = 0.824).

**Table 3:** Concordance between CNB and OEB for receptor status and Ki67 results

**CNB**	**OEB**		**Concordance rate (%)**	**Kappa**	**P value**
	**Negative**	**Positive**			
**ER**			93.6	**0.827**	**<0.001**
Negative	63	4			
Positive	15	216			
**PgR**			85.9	**0.704**	**<0.001**
Negative	95	25			
Positive	17	161			
**HER2**			96.3	**0.894**	**<0.001**
Negative	226	7			
Positive	4	61			
**Ki67**	<14%	≥14%	79.5	**0.545**	**<0.001**
<14%	70	42			
≥14%	19	167			
**HR**			93.6	**0.824**	**<0.001**
Negative	61	5			
Positive	14	218			

*Abbreviation*: CNB, core needle biopsy; OEB, open excision biopsy; ER, estrogen receptor; PgR, progesterone receptor; HR, hormonal receptor.

More Ki67 high expression tumors were detected on OEB samples compared with those on CNB samples (κ = 0.545). We further evaluated whether tumor size, ER, PgR, HER2, and grade status had an effect on Ki67 performance between CNB and OEB. 14% cutoff value was used to define Ki67 high/low expression. ER, PgR, HER2, and grade were detected on CNB samples. There was no concordance rate difference between T_1_ and T_2_ tumors, 77.2% in smaller tumors (T_1_ tumors, n = 123) and 83.8% in larger tumors (T_2_ tumors, n = 160), with κ value of 0.530 and 0.604, respectively. Also, HER2 status had no impact on Ki67 detecting accurate. However, Ki67 concordance rate was much higher in ER negative tumors compared with ER positive tumors (92.5% vs. 76.2%, *P* = 0.003). 60 patients with ER negative and high Ki67 diseases had 100% Ki67 concordance rate between CNB and OEB. Moreover, patients with PgR negative, or grade 3 tumors had a better agreement using CNB to detect Ki67 status than those with PgR positive or grade 1–2 diseases, with *P* value 0.012 and 0.006, respectively.

### Comparison of CNB with OEB for molecular subtypes

Table 4 shows concordance rates for molecular subtypes between CNB and OEB. Using 14% as the Ki67 cutoff value for determining Luminal A and B in HR+/HER2- disease, 32.6% of patients were classified as Luminal A in the CNB samples compared with 26.8% in the surgical specimen. For the remaining patients, 45.4%, 13.1% and 9.1% of cases were respectively classified as Luminal B, triple negative, and HER2 positive diseases using CNB specimens. The concordance rate for detecting these four molecular subtypes was 77.2% between CNB and OEB samples, which also demonstrated as good agreement (κ = 0.658). There were only 2 of the 39 triple negative patients classified as other subtypes on the subsequent surgical specimen. Furthermore, if we subdivided the Luminal B subtype as Luminal B-HER2- and Luminal-HER2+ subtypes according to HER2 status, a similar concordance rate and agreement status was also found (Table 4).

**Table 4:** Concordance between CNB and OEB for molecular subtypes

**CNB**	**OEB**					**Concordance rate (%)**	**Kappa**
							**(P value)**
**Using Ki67 = 14% as cutoff value for determining Luminal A and B in HR+/HER2- diseases**							
**4 Subtypes**	Luminal A	Luminal B		TNBC	HER2+	**77.2**	**0.658 (<0.001)**
Luminal A	65	32		0	0		
Luminal B	15	106		7	7		
TNBC	0	1		37	1		
HER2+	0	4		1	22		
**5 Subtypes**	Luminal A	Luminal B		TNBC	HER2+	**75.8**	**0.679 (<0.001)**
		HER2-	HER2+				
Luminal A	65	31	1	0	0		
Luminal B							
HER2-	14	72	3	7	1		
HER2+	1	1	30	0	6		
TNBC	1	0	0	37	1		
HER2+	0	0	4	1	22		
**Using Ki67 = 20% as cutoff value for determining Luminal A and B in HR+/HER2- diseases**							
**4 Subtypes**	Luminal A	Luminal B		TNBC	HER2+	**79.2**	**0.692 (<0.001)**
Luminal A	110	31		0	0		
Luminal B	10	67		7	7		
TNBC	1	0		37	1		
HER2+	0	4		1	22		
**5 Subtypes**	Luminal A	Luminal B		TNBC	HER2+	**78.2**	**0.699 (<0.001)**
		HER2-	HER2+				
Luminal A	110	29	2	0	0		
Luminal B							
HER2-	9	34	2	7	1		
HER2+	1	1	30	0	6		
TNBC	1	0	0	37	1		
HER2+	0	0	4	1	22		

*Abbreviation*: CNB, core needle biopsy; OEB, open excision biopsy; HR, hormonal receptor; TNBC, triple negative breast cancer.

To be more convenient for our clinical practice, we used 20% as Ki67 cutoff value for determining Luminal A and B subtypes in HR+/HER2- diseases, which was also the mean value in HR+/HER2- patients and median value for the whole population in CNB samples. There were 47.3% of the cases classified as Luminal A subtype in the CNB samples. The overall concordance rates were 79.2% and 78.2% in terms of the four and five molecular subtype classification, respectively. The κ values for these two categories were 0.692 and 0.699, which were also regarded as good agreement (Table 4). However, using a cut-point of Ki67 either 14% or 20% for both specimens, there will be about 14% of HR+/HER2- specimens would be classified as Luminal A on CNB and Luminal B on OEB, indicating Ki67 testing should be repeated in OEB samples.

## Discussion

To our knowledge, this is the first study to evaluate the concordance of molecular subtypes between CNB and subsequent OEB samples in large series of breast cancer patients. In the present study, good agreement was demonstrated in evaluating molecular subtypes as well as ER, PgR and HER2 status between CNB and OEB (κ > 0.6). Although, Ki67 expression was found to be slightly higher in the OEB samples.

Concordance rates of 93.6% for ER, and 85.9% for PgR showed a good correlation with these biomarkers between CNB and OEB, similar to other studies, although the ER concordance rate was relatively higher than with PgR [9,14]. The main explanation may be poorer fixation of OEB compared with CNB specimens, including delayed fixation, under-fixation, and over-fixation with formalin prior to IHC analysis, because the PgR test seems to require a higher preparation quality than an ER test [10,15]. Another reason could be more heterogeneous distribution within the tumor for PgR compared with ER detection [16].

In terms of HER2 examination, a 96.3% concordance rate after adding FISH testing showed that detecting HER2 on CNB samples was as sensitive in predicting HER2 status as OEB. Previous studies have reported concordance rate between CNB and OEB for HER2 examination to be about 90%. However, one study reported a false positive rate of IHC testing on CNB samples as high as 19.3% [17]. A recent meta-analysis showed that the sensitivity and specificity of HER2 status evaluation of CNB was 81% and 89%, respectively, with the HER2 positivity definition as IHC 2+ or 3+ or FISH+. However, the specificity of HER2 detection in CNB would be improved with a very low false positive rate (specificity 98%) using a HER2 positivity definition as IHC 3+ or FISH + [4]. In our cohort, we carried out all FISH testing in IHC HER2 2+ cases, according to the ASCO/ACP HER2 detection recommendation, most likely explain our high concordance rate.

Ki67 antigen has been used to evaluate the proliferative activity of breast cancer for several decades, and a meta-analysis has shown that high Ki67 expression confers a higher risk of relapse and a worse survival [18]. There was an increasing debate about the lack of standardization of Ki67 pathological interpretation and standard cutoff value for Ki67 high expression. Published studies have used various Ki67 cutoff value such as mean, median, the optimal cut-off value or arbitrary values [18]. In the current study, we used 20% (mean value in ER+/HER2- tumors and median value for the whole patients on CNB) as another Ki67 cutoff value (14%) for determining Luminal A and B subtypes in HR+/HER2- diseases. Breast Cancer International Research Group (BCIRG) 001 trial subgroup analysis showed that Ki67 IHC results, whose cutoff value was 14%, can define which ER+/HER2- tumors can get more benefit from adjuvant docetaxel treatment [19]. Moreover, in PACS 01 trial, ER positive breast cancer patients with Ki67 ≥ 20% were more sensitive to docetaxel treatment in the adjuvant setting [20]. In patients with advanced breast cancer, higher Ki67 levels have been significantly associated with decreased time to aromatase inhibitor treatment failure [21]. The comparison of baseline Ki67 labeling index and post-treatment level would enhance the informative value of the test in patients received preoperative endocrine therapy [22]. Gene expression profiling has revealed that the Ki-67 gene seems to play an important role in several “proliferation signatures” and can be assessed by the Ki67 index [23]. Furthermore, Ki67 is a key selected gene in the Oncotype DX^TM^ assay, which can be used to predict the outcome and chemotherapy sensitivity in ER+/HER2- tumors [24,25]. Thus, the 2011 St. Gallen breast cancer consensus recommended that proliferation markers, such as Ki67, can be applied to classify breast cancer into different Luminal subtypes, guiding further treatment [6]. In our study, we found moderate agreement (κ = 0.545) and a slightly higher Ki67 expression in OEB samples compared with CNB samples, with the mean Ki67 expression values of 29.3% and 26.8%, respectively. A major reason for this Ki67 expression difference may be due to sampling error and tumor heterogeneity, as CNB might not reflect the true status of the entire tumor [12]. However, we found no improvement in Ki67 evaluation in smaller compared with larger tumors. There is literature suggesting that four cores can provide sufficient tumor for biomarker testing and good diagnostic accuracy, meaning that Ki67 evaluation might improve with increasing number of cores [26]. However, the level of concordance between the CNB and OEB improved only slightly with increasing number of core passes, but reaching a plateau after 6 or more core passes [27]. In addition, the concordance rates were much higher for ER, PgR, and HER2 than Ki67, which again can be explained by more heterogeneous distribution within the tumor for Ki67, especially in ER/PR positive or grade 1–2 tumors. A comparison of immunocytochemical assays for Ki67 and other biologic variables on preoperative fine-needle aspirates with OBE results showed the concordance between cytology and histology was the lowest for Ki67 evaluation: 89% for ER, 78% for PgR, 79% for p53, and 70% for Ki67, respectively [28]. In one study, Ki67 did not discriminate different biological subtypes of disease with distinct clinical courses rather describe the composition of the mixture of cells in the tumor, which also reflected that the heterogeneity of breast cancer might contribute to Ki67 scoring inconsistence [29]. Greer et al. compared the Ki67 expression between CNB and OEB by IHC and showed a concordance rate of 73% with a κ value of 0.48, similar with our result [27]. Our data indicate that, due to heterogeneous distribution of the Ki67 antigen, CNB may not adequately represent its true biologic profile and Ki67 should be detected on both CNB and OEB in order to avoid misclassifying tumor subtypes and omission of life-saving systemic therapy, especially on HR+/HER2- tumors.

Breast cancer can no longer be considered as a single disease [5,30]. Molecular subtypes can be defined by microarray testing and this classification approximated using IHC results of ER, PgR, HER2 and other proliferation biomarkers. In order to make an appropriate individualized therapeutic strategy, management of breast cancer according to these distinct subtypes is required. CNB is being increasingly used for breast cancer diagnosis and translational research. However, there has been no large published data about the agreement of these molecular subtypes between CNB and OEB in breast cancer. In the current study, with a large series of breast cancer patients, we found good agreement in evaluating molecular subtypes on CNB compared with those on OEB samples (κ = 0.658). Furthermore, a high concordance rate was also detected if by subdividing Luminal B subtype into Luminal B-HER2- and Luminal-HER2+ subtypes. However, approximately 14% of HR+/HER2- specimens would be classified as Luminal A on CNB and Luminal B on OEB, thus depriving these patients of potentially helpful chemotherapy. In summary, our results show a high concordance rate and good agreement between CNB and OEB in the distinction between Luminal and non-Luminal molecular subtypes. However, the differentiation of Luminal A from Luminal B in HR+/HER2- patients is less accurate due to intra-tumoral heterogeneity of Ki67.

## Conclusion

CNB had good agreement in evaluating molecular subtypes as well as ER, PgR, and HER2 status in breast cancer. Due to inherent Ki67 heterogeneity ER+/HER2- tumors, distinguishing Luminal A from Luminal B is less accurate; thus Ki67 ought to be examined both on CNB and OEB samples, especially in HR+/HER2- tumors. Our findings support the recommendation that CNBis considered the initial procedure to assess molecular subtypes and receptor status in invasive breast cancer.

## Competing interests

The authors declare that they have no competing interests.

## Authors’ contributions

XSC, YFL and KWS carried out the conception and design of the study. JHW, WGC and KWS participated in the design and administrative support for the study. XCF and XLJ carried out the histology and IHC analysis. LS, YM, and SJZ carried out the collection and assembly of data. JYW, OH, and YY participated in the data analysis and interpretation of the study, drafted the manuscript. All authors read and approved the final manuscript.

## Pre-publication history

The pre-publication history for this paper can be accessed here:

http://www.biomedcentral.com/1471-2407/13/390/prepub
